# 

**Published:** 1906-09-15

**Authors:** 


					the hospital.
Sept. 15xh,1906.
TALr
WHICH ,
fK? lOnfi fSubscription^Terms"| DDlf?c THRFFPFIMF
as So rt Western Infirmam
NO. 1,043. Vol. XL. [aNewTpfpen] SaTUBDAY,
Skpt. 15th, 1906.
rBnbaseei^i432TermB] PRICE THREEPENCE

				

